# Pertussis Prevalence and Its Determinants among Children with Persistent Cough in Urban Uganda

**DOI:** 10.1371/journal.pone.0123240

**Published:** 2015-04-15

**Authors:** Vincent Kayina, Samuel Kyobe, Fred A. Katabazi, Edgar Kigozi, Moses Okee, Beatrice Odongkara, Harriet M. Babikako, Christopher C. Whalen, Moses L. Joloba, Philippa M. Musoke, Ezekiel Mupere

**Affiliations:** 1 Department of Paediatrics & Child Health, College of Health Sciences, Makerere University, Kampala, Uganda; 2 Department of Medical Microbiology, College of Health Sciences, Makerere University, Kampala, Uganda; 3 Department of Paediatrics, School of Medicine, Gulu University, Gulu, Uganda; 4 Child Health and Development Center, College of Health Sciences, Makerere University, Kampala, Uganda; 5 Department of Epidemiology & Biostatistics, College of Public Health, University of Georgia, Athens, United States of America; Universidad Nacional de La Plata., ARGENTINA

## Abstract

**Background:**

We determined prevalence of pertussis infection and its associated host and environmental factors to generate information that would guide strategies for disease control.

**Methods:**

In a cross-sectional study, 449 children aged 3 months to 12 years with persistent cough lasting ≥14 days were enrolled and evaluated for pertussis using DNA polymerase chain reaction (PCR) and ELISA serology tests.

**Results:**

Pertussis prevalence was 67 (15% (95% Confidence Interval (CI): 12–18)) and 81 (20% (95% CI: 16–24)) by PCR and ELISA, respectively among 449 participating children. The prevalence was highest in children with >59 months of age despite high vaccination coverage of 94% in this age group. Study demographic and clinical characteristics were similar between pertussis and non-pertussis cases. Of the 449 children, 133 (30%) had a coughing household member and 316 (70%) did not. Among 133 children that had a coughing household member, sex of child, sharing bed with a coughing household member and having a coughing individual in the neighborhood were factors associated with pertussis. Children that had shared a bed with a coughing household individual had seven-fold likelihood of having pertussis compared to children that did not (odds ratio (OR) 7.16 (95% CI: 1.24–41.44)). Among the 316 children that did not have a coughing household member, age <23 months, having or contact with a coughing individual in neighborhood, a residence with one room, and having a caretaker with >40 years of age were the factors associated with pertussis. Age <23months was three times more likely to be associated with pertussis compared to age 24–59 months (OR 2.97 (95% CI: 1.07–8.28)).

**Conclusion:**

Findings suggest high prevalence of pertussis among children with persistent cough at a health facility and it was marked in children >59 months of age, suggesting the possibility of waning immunity. The factors associated with pertussis varied by presence or absence of a coughing household member.

## Introduction

The control of pertussis or whooping cough is of public health concern worldwide, because it is estimated that over 10 million cases and as many as 400,000 pertussis-related deaths occur annually, with 90% of the burden in infants from developing countries [1,2,3]. However, the optimum strategies to improve control of pertussis remain uncertain as robust data for disease incidence and surveillance are sparse particularly in developing countries [2]. Pertussis affects all age groups and can occur in previously immunized or infected individuals [4,5]. The introduction of mass vaccination of children in the mid-20^th^ century decreased the incidence of pertussis significantly; however, pertussis has remained endemic because available vaccines prevent disease but to a lesser extent infection and transmission [1,6,7]. The infection continues to kill young children and even in countries with strong vaccination programs clinicians miss the diagnosis [1,3]. Over the last two decades there has been a resurgence of pertussis disease in well-vaccinated populations worldwide, largely due to rapid loss of protective immunity and pathogen adaptation [6,7]. The resurgence has been accompanied by a shift to older age groups, raising concerns about household transmission to vulnerable infants and the need for reinforcement of vaccination strategies.

In Uganda, pertussis vaccination was introduced in 1974 by the National Expanded Program in Immunization (UNEPI). The UNEPI provides three doses of whole cell pertussis vaccine at 6, 10, and 14 weeks of age in combination with diphtheria, tetanus, hepatitis B and hemophilus influenza type b with no booster doses. The current vaccination coverage for 3^rd^ dose of pertussis is at 74% [8]; however, its impact on the epidemiology of pertussis disease in Uganda is limited to guide strategies for disease control. Further, due to limited patient and clinician awareness, and lack of access to sensitive diagnostic methods [1,3], existing data are challenged with underreporting and misdiagnosis. Therefore there are few data on the burden of pertussis in Africa to systematically understand the impact of the immunization program on pertussis morbidity and mortality.

To fill this existing gap in knowledge, we conducted a cross-sectional study that established the burden of pertussis infection among children presenting with persistent cough at a national referral teaching hospital and the potential modifiable host, caretaker and environmental factors that may be associated with the infection in urban Uganda. The findings provide insight into future prevention and control strategies, and raise awareness of clinicians as regards the existence of pertussis infection.

## Materials and Methods

### Design and setting

In a cross-sectional study design, 449 children in the age range of 2 months to 12 years presenting with persistent cough lasting two or more weeks were conveniently and consecutively enrolled and screened for pertussis infection. The study was conducted at the Makerere-Mulago hospital Acute Care Unit (ACU) and the Assessment Center, located five meters apart between July and December of 2013. Mulago hospital is the national referral and largest University teaching public hospital for Makerere University in Uganda with a bed capacity of 1,500. The hospital receives mostly self-referred patients residing in Kampala (the capital city), and patients residing in the surrounding districts. The hospital also receives referred patients countrywide from any other health facilities. The ACU is the emergency ward for children whereas the assessment center provides general medical outpatient services for over 120 children of all ages daily. The ACU admits all children (aged 0–12 years) with medical conditions.

### Ethical considerations and human subjection protection

The study protocol was approved by the School of Biomedical Sciences Research and Ethics Committee at College of Sciences, Makerere University (SBREC MakCHS) and cleared by the Uganda National Council for Science and Technology (UNCST). Individual written consent was obtained from the caretakers on behalf of the participating children. Consent forms were translated into the local language, and written consent was obtained by thumb print for caretakers who were unable to write. This consent procedure was approved by the research ethics committee. All study participants received care and treatment according to the care and treatment protocols for Mulago hospital.

### Laboratory Diagnosis of Pertussis

Diagnosis of pertussis infection was performed using polymerase chain reaction (PCR) and ELISA antibody tests. Whole blood and nasopharyngeal swabs were collected from all participating children for ELISA and PCR assays, respectively and were stored at -80°C and analyzed as a batch. Pertussis diagnosis using PCR was carried out by detecting *Bordetella pertussis* and *B*. *parapertussis*. An in-house PCR assay targeting *PTXS1* portion of *B*. *pertussis* toxin gene common to *B*. *pertussis*, *B*. *parapertussis* and *B*. *bronchiseptica;* and *IS10002* unique to *B*. *pertussis* and *B*. *parapertussis* was used as recommended by Centers for Disease Control and Prevention [9]. The DNA was extracted from nasopharyngeal swabs using Epicenter DNA Extraction Kit (Epicenter Biotechnologies, USA) following the manufacturer’s instructions. The quantity and quality of the DNA was assessed using NanoDrop spectrophotometer 2000c (Thermo scientific, USA). The DNA of *B*. *pertussis* strain ATCC9797D-5 and PCR water were used as positive and negative controls respectively. To rule PCR Inhibition, Human beta-actin gene was amplified in the entire sample. A sample scored as *B*. *pertussis* and/or *B*. *parapertussis* positive if it had specific amplification for *PTSX-1*(55bp) and *IS1002* (100bp).

The ELISA antibody test for IgG against pertussis toxin was also performed using the *B*. *pertussis* IgG-PT ELISA kit (Statens Serum Institut Diagnostica, Denmark), as per the manufacturer’s instructions. The ELISA antibody test was used to confirm evidence of recent infection at a cutoff of ≥100 IU/ml among 408 samples [10]. Serology was not performed for 41 participants because samples lysed before tests were used.

### Independent variables

All participating children were assessed for the following possible confounders and effect modifiers to evaluate for host, caretaker and environmental factors associated with pertussis using a standardized study tool and administered by a trained health worker: socio-demographics such as age and sex; status for pertussis vaccination; HIV status; caretaker variables such as age, level of education and alcohol intake; household variables such as household size, household number of rooms, presence of windows, type and location of residence, sharing of bed and bed room with a household member who had persistent cough (hereafter referred to as coughing household member); presence and contact with a community member who had persistent cough in the immediate neighborhood (hereafter referred to as coughing individual in the neighborhood). HIV testing was available as routine service for children presenting at the hospital using HIV DNA PCR and HIV rapid tests for children below or over 18 months, respectively.

### Statistical Considerations

Sample size estimation was based on attaining adequate precision to establish prevalence of pertussis among children under five years and among children aged five or more years that had presented with persistent cough at Mulago national referral teaching hospital. A sample size of 217 was estimated per age group, assuming a prevalence of 17% among children with persistent cough based on previous data [11], a tolerable error margin of the estimate of 5% and a type I error of 5%. A minimum sample size of 434 children with persistent cough was therefore needed for enrolment into the study.

In the analytic strategy, we performed descriptive statistics for all study variables using proportions for binary and means for continuous variables. Proportions for prevalence of pertussis were computed with 95% confidence interval (CI). Proportions were compared using chi-square and means with student t-test after testing variables for assumptions of normality. We performed stratified analysis to explore potential for confounding and effect modification among study variables. We performed a series of univariate and multivariable logistic regression analyses to establish associations between the host, caretaker or environmental factors and pertussis determined by PCR as a primary outcome variable. The negative two log likelihood ratio tests were used to test the best parsimonious models. Two way interactions between co-variables were evaluated; significant interactions were demonstrated between contact with a coughing household member and 24–59 months age of participating children on pertussis infection. Therefore, separate univariate and multivariable logistic regression models were fit according to presence or absence of a coughing household member.

## Results

**Fig 1:**
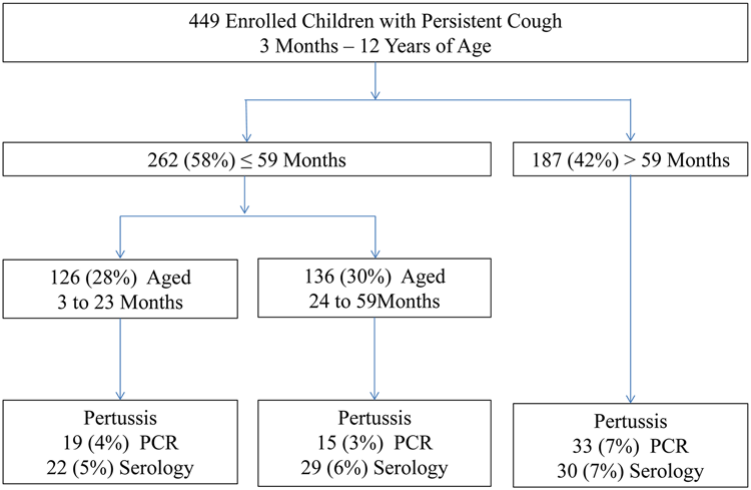
Study Flow Chart of Enrolled Participants According to Age at Mulago National Referral Teaching Hospital in Kampala, Uganda (n = 449).

Of the 449 children who were enrolled with persistent cough of 14 days or more, over half (262/449) of the study participants were children below five years (Fig 1). The mean age among 449 children who were included in the analysis was 49 ± 34 months with a range of 3 months to 12 years. Most (398/418) were outpatients and (411/449) fully immunized (Table 1). A third (133/449) of the participating children had contact with a coughing household member and 193/421 with a coughing individual in the neighborhood. Nearly a quarter (101/435) of the participating children were sleeping in the same room with a coughing household member, and 51/416 were sleeping in the same bed. Nine percent (38/442) of households for the participating children had poor ventilation with no windows.

**Table 1:** Serology for pertussis, host and environmental factors associated with confirmed pertussis by PCR among 449 children at a national referral teaching hospital in Uganda.

Characteristic and Number of Responding Participants	Distributions	PCR Confirmed Pertussis		p-value
		No (n = 382)	Yes (n = 67)	
	n (%)	n (%)	n (%)	
Pertussis by serology (n = 408)				
Negative	327 (80)	279 (80)	48 (83)	
Positive	81 (20)	71 (20)	10 (17)	0.590
Age group, months (n = 449)				
3–23	126 (28)	107 (28)	19 (28)	0.953
24–59	136 (30)	121 (32)	15 (22)	
>59	187 (42)	154 (40)	33 (49)	0.172
Sex (n = 441)				
Female	212 (48)	179 (48)	33 (50)	
Male	229 (52)	196 (52)	33 (50)	0.756
District of residence (n = 449)				
Kampala city	341 (76)	51 (76)	290 (76)	
Outside Kampala city	108 (24)	16 (24)	92 (24)	0.971
Category (n = 418)				
Inpatient	20 (5)	14 (4)	6 (10)	
Outpatient	398 (95)	343 (96)	55 (90)	0.061
Child HIV status (n = 449)				
Negative	433 (96)	370 (97)	63 (94)	
Positive	16 (4)	12 (3)	4 (6)	0.258
Immunization (n = 449)				
Complete	411 (92)	350 (92)	61 (91)	
Incomplete	38 (8)	32 (8)	6 (9)	0.875
Coughing household member (n = 449)				
No	316 (70)	266 (70)	50 (75)	0.410
Yes	133 (30)	116 (30)	17 (25)	
sleep in same room with coughing household member (n = 435)				
No	334 (77)	281 (76)	53 (82)	0.327
Yes	101 (23)	89 (24)	12 (18)	
Sleep in same bed with coughing household member (n = 416)				
No	365 (88)	304 (87)	61 (94)	0.112
Yes	51 (12)	47 (13)	4 (6)	
Coughing individual in neighborhood (n = 449)				
No	247 (55)	216 (57)	31 (46)	0.121
Yes	202 (45)	166 (43)	36 (54)	
Contact with coughing individual in neighborhood (n = 421)				
No	228 (54)	195 (55)	33 (50)	0.262
Yes	193 (46)	160 (45)	33 (50)	
Household with windows (n = 442)				
No	38 (9)	29 (8)	9 (14)	0.118
Yes	404(91)	347 (92)	57 (86)	
Number of Rooms in household (n = 448)				
>1	237 (53)	198 (52)	39 (58)	0.346
1	211 (47)	183 (48)	28 (42)	
Number of people in household (n = 446)				
≤5 people	344 (77)	290 (76)	54 (82)	0.328
>5 people	102 (23)	90 (24)	12 (18)	
Type of household (n = 449)				
Other	198 (44)	167 (44)	31 (46)	0.698
Muzigo	251 (56)	215 (56)	36 (54)	
Type of neighborhood (n = 449)				
Other	281 (63)	246 (64)	35 (52)	0.060
Slum	168 (37)	136 (36)	32 (48)	
Age of caretaker, years (n = 448)				
<25	123 (27)	109 (29)	14 (21)	
25–40	286 (64)	243 (64)	43 (64)	0.929
>40	39 (9)	29 (7)	10 (15)	0.054
Caretaker takes alcohol (n = 448)				
No	42 (9)	39 (10)	3 (4)	0.148
Yes	406 (91)	342 (90)	64 (96)	

PCR = Polymerase chain reaction

Among 133 children that had household individual with persistent cough, 101 (76%) of the children slept in the same room and 51 (40%) in the same bed with the coughing household member, 76 (57%) had a coughing individual in the neighborhood, and 15 (12%) had no windows on the house of residence. Among 316 children that had no coughing household member, 122 (42%) had contact with a coughing individual in the neighborhood, 149 (47%) had households with only one room, and 26 (8%) of their caretakers were greater than 40 years of age.

**Fig 2:**
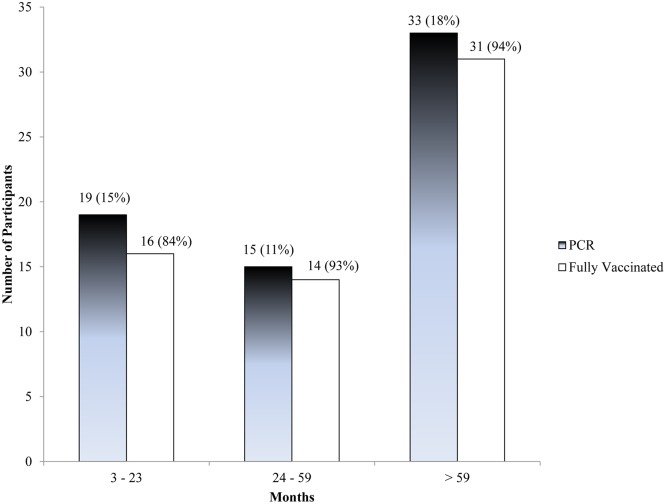
Age-specific Prevalence of Pertussis Infection and Vaccination Coverage among 3 Months to 12 Year Old Children Presenting at Mulago National Referral Teaching Hospital in Kampala, Uganda (n = 449). PCR = Polymerase chain reaction; for ‘Fully vaccinated’ represent the percentage of cases in that age group which was vaccinated.

The prevalence of pertussis according to PCR tests among 449 children with persistent cough was 67 (15% (95% CI: 12–18)), and by ELISA serological tests among 408 children was 81 (20% (95% CI: 16–24)). There was modest agreement between PCR and serology results of 71% (289/408) and poor kappa statistic of -0.03 (McNemar’s test, χ^2^ = 4.45, p = 0.035) regardless of age category. The prevalence as measured by PCR was highest in children aged >59 months despite similar high age-specific vaccination level (Fig 2). Nearly half (33/67) of the children diagnosed by PCR were above five years of age. Moreover, 94% (31/33) of the children diagnosed by PCR above five years were fully vaccinated (Fig 2).

Pertussis was detected in children regardless of whether or not they had a coughing member in the household or neighborhood. Of the 316 participating children in households with no coughing individual, 50 (16%) and 57 (19%) had pertussis diagnosed by PCR and serology, respectively. Similarly, of the 133 that had coughing household member, 17 (13%) and 24 (21%) had pertussis diagnosed by PCR and serology, respectively.

We found no significant differences when host and environmental factors were compared between children diagnosed with and without pertussis by PCR. Of note; however, a large number 55 (90%) of children diagnosed with pertussis were outpatients, 61 (91%) had complete vaccination, 36 (54%) had coughing individual in the neighborhood, and male to female ratio was 1:1 (Table 1). The clinical and laboratory features were comparable between children with or without pertussis diagnosed by PCR (S1 Table). For example, no children (0%) with pertussis had cough with a whoop compared to 3 (1%) among children without pertussis (p = 1.00). Among 67 children with pertussis, 3 (4%) had conjunctival injection compared to 15 (4%) among 382 children without pertussis (p = 0.832).

There was a significant interaction between age of children in the range of 24 to 59 months and history of contact with a coughing household member on detection of pertussis (p = 0.047). Among children that had history of contact with a coughing household member, we found sex, category of being inpatient or outpatient, sleeping in bed with a coughing household member, and having a coughing individual in the neighborhood to be factors associated with pertussis after adjusting for age, vaccination status, presence of household windows, and alcohol intake status of caretaker (Table 2). Children that had slept in bed with the coughing household member were seven times more likely to have pertussis compared to children that did not (odds ratio (OR) 7.16 (95% CI: 1.24–41.44); Table 2). Further, children that had a coughing individual in the neighborhood were also seven times more likely to have pertussis compared to those that did not (OR 7.23 (95% CI: 1.45–36.01)).

**Table 2:** Host and environmental factors associated with confirmed pertussis by PCR among 449 children at a national referral teaching hospital in Uganda stratified according to status of a coughing household member.

	Overall Model		Stratified Models	
Characteristic			Coughing Household Member Absent	Coughing Household Member Present
	Pertussis Cases (n = 67)	Odds Ratio (95% CI)	Odds Ratio (95% CI)	Odds Ratio (95% CI)
Age group, months	n (%)			
3–23	19/126 (15)	1.88 (0.83–4.26)	2.97 (**1.07–8.28**)	0.42 (0.08–2.35)
24–59	15/136 (11)	1	1	1
>59	33/187 (18)	1.38 (0.66–2.90)	1.99 (0.75–5.30)	0.45 (0.11–1.86)
Sex				
Female	33/212 (16)	1	1	1
Male	33/229 (14)	0.96 (0.54–1.71)		4.87 (**1.28–18.55**)
Immunization				
Complete	61/411 (15)	1	1	1
Incomplete	6/38 (16)	1.14 (0.36–3.56)	0.69 (0.18–2.60)	3.90 (0.17–89.40)
Category				
Inpatient	6/20 (30)	1	1	1
Outpatient	55/398 (14)	2.41 (0.77–7.56)	2.95 (0.80–10.83)	25.77 (**1.81–367.52**)
Sleep in same bed with coughing household member				
No	61/365 (17)	1	1	1
Yes	4/51 (8)	5.29 (**1.20–23.29**)		7.16 (**1.24–41.44**)
Coughing individual in neighborhood				
No	31/247 (13)	1	1	1
Yes	36/202 (18)	3.29 (0.56–19.45)	23.65 (**2.13–262.81**)	7.23 (**1.45–36.01**)
Contact with coughing individual in neighborhood				
No	33/228 (14)	1	1	1
Yes	33/193 (17)	2.73 (0.46–16.15)	29.62 (**2.57–340.87**)	
Household windows present				
Yes	57/404 (14)	1	1	1
No	9/38 (24)	2.83 (**1.10–7.25**)	1.75 (0.51–6.09)	2.44 (0.45–13.12)
Rooms in household				
>1	28/211 (13)	1	1	1
1	39/237 (16)	1.89 (**1.00–3.57**)	2.23 (**1.04–4.77**)	
Household size				
≤5 people	12/102 (12)	1	1	1
>5 people	54/344 (16)	0.59 (0.28–1.24)	0.47 (0.19–1.14)	
Type of neighborhood				
Other	35/281 (12)	1	1	1
Slum	32/168 (19)	1.70 (0.94–3.08)	1.81 (0.87–3.77)	
Age of caretaker, years				
<25	14/123 (11)	1	1	1
25–40	43/286 (15)	1.68 (0.79–3.56)	2.14 (0.88–5.21)	
>40	10/39 (26)	3.59 (**1.17–11.06**)	7.74 (**2.04–29.36**)	
Caretaker takes alcohol				
No	64/342 (16)	1	1	1
Yes	3/42 (7)	2.37 (0.65–8.65)	6.83 (0.85–54.63)	6.29 (0.80–49.43)

PCR = Polymerase chain reaction; CI = Confidence Interval

Among children that had no history of contact with a coughing household member, we found young age <24 months, having or contact with a coughing individual in the neighborhood, residing in household with one room, and having a caretaker aged >40 years to be factors associated with pertussis after adjusting for age groups ≥24 months, being outpatient, presence of household windows, household number of people, residing in a slum neighborhood, and alcohol intake status of caretaker (Table 2). For example, young children <24 months of age were three times more likely to have pertussis compared to children aged 24 to 59 months (OR 2.97 (95% CI: 1.07–8.28)); (Table 2). Children that had exposure to a coughing individual in the neighborhood were nearly 30 times more likely to have pertussis compared to children that had no exposure to the coughing individual in the neighborhood (OR 29.62 (2.57–340.87)). Further, children that were residing in households with only one room were two times more likely of having pertussis compared to children that were in households with more than one room (OR 2.23 (1.04–4.77)).

## Discussion

In a cross-sectional study of 449 children that presented with persistent cough lasting 14 or more days, a high prevalence of pertussis infection was found. The associated factors for pertussis infection were influenced or varied by presence or absence of a coughing household member. Immunization status against pertussis was high and comparable among subgroups of participating children. Similarly, the clinical features among children with or without pertussis did not differ; suggesting that persistent cough in children is non-specific for pertussis. It may also be difficult to differentiate between pertussis and other disease conditions such as tuberculosis that present with persistent cough.

Our study findings suggest that community acquired pertussis is prevalent among children that present with persistent cough despite the high pertussis vaccination coverage with three doses of whole cell vaccine in infancy; and in a program with no booster doses and no adolescent or adult vaccination schedules. These findings are consistent with the few studies that have demonstrated that pertussis is endemic with recurrent outbreaks and could be re-emerging in Africa [12,13,14,15]. Zouari A et al reported a pertussis prevalence of 20% using PCR [12] in 599 Tunisian hospitalized infants who were recruited between 2007 and 2011. The lower prevalence of 15% in our study compared to the study in Tunisia may be explained by variations in study populations. Our study included children with a wide age range (3 months to 12 years), and most (>90%) had completed their primary vaccination compared to Tunisian infants who were aged <12 months and had not completed the three doses of pertussis vaccination.

The high percentage of pertussis cases among children >59 months in our study population in the face of high vaccination coverage, leads us to believe that rapid waning of vaccine-induced and natural immunity is a possible explanation. Waning immunity has been described as the key possible cause for the resurgence of pertussis in developed countries [16,17,18,19]. We envisage that waning of immunity may be faster in African populations because of high prevalence of malnutrition and HIV/AIDS which may cause ineffective immunological memory mechanisms and inefficient secondary immune response [6]. However, very few of the study children were HIV infected.

Our study findings provide insight into the possible drivers for pertussis transmission. Host characteristics such as male sex and young age less than two years were associated with pertussis. At the household, degree of contact with the potentially infected family member and household factors like single room household appear to be important determinants in household transmission. Our findings are therefore in line with several household contact studies and systematic reviews that revealed over 50% of the sources of childhood pertussis to be at household level [20,21,22,23,24,25]. The close contact at household level provides high risk of exposure to the infective pertussis dose for transmission. However, it is not clear from our findings why male sex was associated with increased likelihood for infection if the participating child had had contact with a coughing household family member. Our findings also provide clues to possible non-household environmental factors that could be responsible for the over 30% pertussis infection seen in children with no identifiable source or exposure status [20,21,22] such as casual contact with a coughing individual in the neighborhood, and caretakers with age >40 years.

Despite the important associations seen in our study, there are caveats to interpretations of our study findings. We employed a cross-sectional design therefore the associations in our study findings are not causal. A prospective study may be needed to confirm our study findings. However, we took care to phrase questions that reflect temporal relationships between potential exposures and the outcome; and our study findings have biological plausibility and consistency of the associations are similar to those that were obtained through prospective studies [20,21,22,23,24,25]. We used a large sample size to evaluate prevalence using recommended laboratory tests and it gave us opportunity to explore interactions between exposures and prevalence. We used self recall for immunization status and other exposure variables. The study was conducted at a tertiary referral center; therefore, there may be referral bias which might have brought sicker children likely to have pertussis and other diseases such as tuberculosis.

In conclusion, we found a high prevalence of pertussis in this cross-sectional study of children with persistent cough lasting 14 or more days at a health facility. The percentage of pertussis cases was highest in children >59 months despite associated high vaccination level suggesting the possibility of waning immunity; however, the potential determinants for the infection among participating children appeared to differ by prior history of contact with a coughing household member or an individual in the neighborhood. Immunization status against pertussis was high and comparable among subgroups of participating children. Our findings support the need to strengthen routine and booster dose immunization to support infants against pertussis morbidity and mortality; for clinicians to screen for pertussis using PCR, a sensitive and specific laboratory test [1,26,27] to institute appropriate medical care and treatment; and for the public to be aware of pertussis existence, its presentation to seek the necessary medical care, and the key drivers for its transmission. Prospective studies should be conducted to confirm the plausibility and consistency for the associations found in the present study.

## Supporting Information

S1 Table Clinical and laboratory characteristics among 449 children with or without confirmed pertussis by PCR. PCR = Polymerase chain reaction
(DOCX)
