# Supplementary material for: Pertussis Prevalence and Its Determinants among Children with Persistent Cough in Urban Uganda
Source: PLoS One. 2015 Apr 15;10(4):e0123240. doi: 10.1371/journal.pone.0123240 (PMC4398436; doi:10.1371/journal.pone.0123240)
Supplement: S1 Table — PCR = Polymerase chain reaction (DOCX) [file pone.0123240.s001.docx]

**S1 Table: Clinical and laboratory characteristics among 449 children with or without confirmed pertussis by PCR**

| **Characteristic** | **PCR Confirmed Pertussis** | |  |
| --- | --- | --- | --- |
|  | No (n=382) | Yes (n=67) | p-value |
| Duration of cough, days (mean STD) | 65 (171) | 65 (137) | 0.972 |
| Cough with whooping, n (%) |  |  |  |
| No | 379 (99) | 67 (100) | 0.467 |
| Yes | 3 (1) | 0 (0) |  |
| Fever |  |  |  |
| No | 162 (42) | 26 (39) | 0.581 |
| Yes | 220 (58) | 41 (61) |  |
| Fever, days (mean STD) | 25 (128) | 13 (10) | 0.191 |
| Conjuctival injection |  |  |  |
| No | 367 (96) | 64 (96) | 0.832 |
| Yes | 15 (4) | 3 (4) |  |
| Conjunctival injection, days (mean STD) | 603 (1262) | 118 (169) | 0.169 |
| Fast breathing |  |  |  |
| No | 349 (91) | 61 (91) | 0.932 |
| Yes | 33 (9) | 6 (9) |  |
| Fast breathing, days (mean STD) | 47 (104) | 21 (14) | 0.547 |
| Grunting |  |  |  |
| No | 370 (97) | 63 (94) | 0.249 |
| Yes | 12 (3) | 4 (6) |  |
| Grunting, days (mean STD) | 167 (322) | 15 (11) | 0.129 |
| Chest indrawing |  |  |  |
| No | 380 (99) | 67 (100) | 0.553 |
| Yes | 2 (1) | 0 (0) |  |
| Chest crackles |  |  |  |
| No | 340 (89) | 62 (93) | 0442 |
| Yes | 40 (11) | 5 (7) |  |
| Vomiting |  |  |  |
| No | 302 (79) | 52 (78) | 0.777 |
| Yes | 80 (21) | 15 (22) |  |
| Vomiting, days (mean STD) | 11 (22) | 18 (19) | 0.255 |
| Post-tussive vomiting |  |  |  |
| No | 304 (80) | 57 (85) | 0.296 |
| Yes | 78 (20) | 10 (15) |  |
| Post-tussive vomiting, days (mean STD) | 16 (24) | 18 (20) | 0.778 |
| Antibiotic use |  |  |  |
| No | 129 (34) | 19 (28) | 0.363 |
| Yes | 250 (66) | 48 (72) |  |
| WBC, (mean STD) | 10.4 (5.9) | 9.9 (4.5) | 0.447 |
| Neutraphils, % (mean STD) | 46 (18) | 46 (17) | 0.798 |
| lymphocytes, % (mean STD) | 38 (17) | 41 (18) | 0.277 |
| Heamoglobin, mg/dl (mean STD) | 11,5 (3.9) | 11.5 (2.5) | 0.883 |

PCR =Polymerase chain reaction
